# Patient characteristics and clinical factors affecting lumpectomy cavity volume: implications for partial breast irradiation

**DOI:** 10.3389/fonc.2023.1118713

**Published:** 2023-05-23

**Authors:** Amy Le, Flora Amy Achiko, LaKeisha Boyd, Mu Shan, Richard C. Zellars, Ryan M. Rhome

**Affiliations:** ^1^ Department of Radiation Oncology, Indiana University School of Medicine, Indianapolis, IN, United States; ^2^ Department of Biostatistics, Indiana University School of Medicine, Indianapolis, IN, United States

**Keywords:** partial breast irradiation (PBI), breast cancer, lumpectomy, race, hypertension, BMI, neoadjuvant chemotherapy, prone

## Abstract

**Introduction:**

Partial breast irradiation (PBI) has increased in utilization, with the postoperative lumpectomy cavity and clips used to guide target volumes. The ideal timing to perform computed tomography (CT)–based treatment planning for this technique is unclear. Prior studies have examined change in volume over time from surgery but not the effect of patient characteristics on lumpectomy cavity volume. We sought to investigate patient and clinical factors that may contribute to larger postsurgical lumpectomy cavities and therefore predict for larger PBI volumes.

**Methods:**

A total of 351 consecutive women with invasive or *in situ* breast cancer underwent planning CT after breast-conserving surgery at a single institution during 2019 and 2020. Lumpectomy cavities were contoured, and volume was retrospectively computed using the treatment planning system. Univariate and multivariate analyses were performed to evaluate the associations between lumpectomy cavity volume and patient and clinical factors.

**Results:**

Median age was 61.0 years (range, 30–91), 23.9% of patients were Black people, 52.1% had hypertension, the median body mass index (BMI) was 30.4 kg/m², 11.4% received neoadjuvant chemotherapy, 32.5% were treated prone, mean interval from surgery to CT simulation was 54.1 days ± 45.9, and mean lumpectomy cavity volume was 42.2 cm^3^ ± 52.0. Longer interval from surgery was significantly associated with smaller lumpectomy cavity volume on univariate analysis, p = 0.048. Race, hypertension, BMI, the receipt of neoadjuvant chemotherapy, and prone position remained significant on multivariate analysis (p < 0.05 for all). Prone position vs. supine, higher BMI, the receipt of neoadjuvant chemotherapy, the presence of hypertension, and race (Black people vs. White people) were associated with larger mean lumpectomy cavity volume.

**Discussion:**

These data may be used to select patients for which longer time to simulation may result in smaller lumpectomy cavity volumes and therefore smaller PBI target volumes. Racial disparity in cavity size is not explained by known confounders and may reflect unmeasured systemic determinants of health. Larger datasets and prospective evaluation would be ideal to confirm these hypotheses.

## 1 Introduction

Adjuvant radiation is typically indicated after breast**-**conserving surgery (BCS) to improve local control, which translates on meta-analysis to survival benefits (1–6). Multiple published studies have clearly established the efficacy of the partial breast irradiation (PBI) technique for adjuvant radiation after BCS. Randomized trials comparing external beam PBI with whole breast radiation (WBRT) have shown no clinically significant difference in survival, regional recurrence, or ipsilateral breast tumor recurrence (IBTR) with median follow**-**up ranging from 5 to greater than 10 years (7–12). Brachytherapy options exist for PBI, but accelerated (twice daily) radiation appears to be associated with worse cosmesis, and brachytherapy has been shown to result in increased IBTR (8, 13).

Variation exists in the application of the PBI technique. For external beam PBI, the postoperative lumpectomy cavity with or without clips is generally used to guide volumes to target the postoperative tumor bed and a margin of adjacent breast tissue. The NSABP B-39/RTOG 0412 protocol specified the lumpectomy excision cavity was outlined based on clear visualization on computed tomography (CT) or with the help of surgical clips if those were placed, and the clinical target volume (CTV) was defined as a 15**-**mm uniform expansion of the lumpectomy cavity, while the planning target volume (PTV) was defined as a uniform 10**-**mm expansion of the CTV. If the lumpectomy cavity could not be delineated clearly or the lumpectomy cavity/whole breast reference volume was >30% based on postoperative CT scan, then the patient was not eligible for study participation (8). Even without potential ineligibility based on the size of the lumpectomy cavity, a larger lumpectomy cavity will lead to larger radiation field sizes. The ideal postsurgical timing to perform CT-based treatment planning for the PBI technique is unclear. Prior studies have examined change in volume over time from surgery and associated clinical factors, with expected decrease in the size of the lumpectomy cavity or seroma volume over the postoperative period (14, 15). One study by Kader et al. demonstrated that seroma volume correlated significantly with the volume of excised breast tissue but not with other clinical characteristics including tumor diameter, surgical re-excision, and chemotherapy use (14). Simulation and treatment at the optimal time for a minimum lumpectomy cavity volume that can still be clearly delineated could increase the proportion of patients eligible for PBI and/or decrease treatment volumes.

The effect of patient characteristics including comorbidities that may affect healing in the postoperative period has not been previously reported. We sought to investigate patient and clinical factors that may contribute to larger postsurgical lumpectomy cavities and therefore would predict for larger PBI volumes.

## 2 Materials and methods

This study involved the secondary use of private information from the electronic medical record and was approved as exempt by the institutional review board. A total of 351 consecutive women with invasive or *in situ* breast cancer underwent CT after BCS as part of the standard planning for PBI or WBRT. CT images were obtained using 5-mm slice thickness, with the scan extending from superior to the suprasternal notch to a minimum of 5 cm below the inframammary fold. The CT images were transferred to the treatment planning system (TPS) (Eclipse, Varian Medical Systems, Palo Alto, CA).

There were 357 total lumpectomy cavities that were contoured per institutional standard at a single institution with four radiation centers during the years 2019 and 2020. In general, the lumpectomy cavity delineation included all related radiopaque surgical clips when present. In addition, contouring the seroma and surgical anatomical changes in conjunction with preoperative imaging, operative note, and pathology report was the standard with or without clips. Six patients had two lumpectomies during the same surgery due to invasive or *in situ* breast cancer in bilateral breasts. One patient had two lumpectomies in separate quadrants of the ipsilateral breast. For patients with multiple lumpectomy cavities contoured, the first record in the dataset was included and the second record was excluded in order to prevent duplicate records for the analysis of patient characteristics, resulting in 351 total included lumpectomy cavities. Contoured volume was retrospectively computed from the TPS.

Clinical data extracted from the patients’ medical records included age at the time of surgery, the body mass index (BMI) at the time of surgery, race, the presence of diabetes mellitus, smoking status, the presence of hypertension, the presence of coronary artery disease, the date of last definitive BCS (including repeat excision), the date of planning CT scan, whether surgical re-excision had been performed, the pathologic maximal diameter of the primary tumor, volume excised from lumpectomy, and additional margins (total volume of excised breast tissue), whether patients had undergone oncoplastic reduction, prior surgery or prior biopsy in ipsilateral breast, neoadjuvant hormone therapy use (including aromatase inhibitor or tamoxifen), neoadjuvant chemotherapy use, and adjuvant chemotherapy use. From the CT simulation in the TPS, the position (supine or prone), the presence of surgical clips placed at time of lumpectomy, and physician-contoured lumpectomy cavity volume were extracted. The institutional preference for positioning is the prone position; however, if the patient has smaller, non-pendulous breasts and/or difficulty remaining in the prone position due to discomfort, then the supine position is used.

The change in lumpectomy cavity volume relative to interval after surgery was calculated with linear regression using the gradient estimation method with a log transform. Since the original data for lumpectomy cavity volume followed a log-normal distribution or approximately so, the log transform was performed to reduce skewness resulting in a near-normal distribution. To evaluate the associations among patient, clinical, and treatment factors on lumpectomy cavity volume, univariate analysis was performed and variates with a p-value < 0.1 were included in multivariate analysis.

## 3 Results

The distribution of patient, clinical, and selected treatment characteristics are summarized in **Tables 1A****, B**. Median age was 61.0 years (range, 30–91). The median BMI was 30.4 kg/m² (first quartile [Q1] to third quartile [Q3], 25.5–35.2). There were 23.9% of patients who were Black people, 71.2% of patients were White people, and 4.8% were other. There were 52.1% of patients who had hypertension, 17.1% had diabetes mellitus, 6.0% had coronary artery disease, 12.0% were current smokers, 29.9% were former smokers, and 58.1% were never smokers. The mean tumor size was 1.3 cm (standard deviation, 1.1).

**Table 1A:** Patient, clinical, and treatment characteristics.

Characteristic	N	Mean ± SD	Median (Q1–Q3)
Age (y) **mean ± standard**	351	60.5 ± 10.6	61.0 (53.0–68.0)
BMI (kg/m^2^) **mean ± standard**	332	31.1 ± 7.3	30.4 (25.5–35.2)
Days from surgery **median (Q1–Q3)**	351	54.1 ± 45.9	37.0 (28.0–57.0)
Lumpectomy volume (cm^3^) **median (Q1 –Q3)**	351	42.2 ± 52.0	26.7 (14.8–53.9)
Total volume excised (cm^3^) **median (Q1 –Q3)**	345	175.7 ± 322.0	89.6 (58.5–161.3)
Tumor size (max dimension of largest tumor, cm) **median (Q1–Q3)**	351	1.3 ± 1.1	1.1 (0.6–1.8)

**Table 1B:** Patient, clinical, and treatment characteristics.

Surgical re-excision	Yes	35 (10.0%)
Oncoplastic reduction	Yes	31 (8.8%)
Prior breast surgery	Yes	18 (5.1%)
Neoadjuvant AI or tamoxifen	Yes	15 (4.3%)
Neoadjuvant chemotherapy	Yes	40 (11.4%)
Adjuvant chemotherapy	Yes	53 (15.1%)
Race	Black people	84 (23.9%)
	Other	17 (4.8%)
	White people	250 (71.2%)
Diabetes mellitus	Yes	60 (17.1%)
Smoking status	Current	42 (12.0%)

All patients underwent BCS, with 10% requiring surgical re-excision, 8.8% had oncoplastic reduction, 5.1% had prior surgery or prior biopsy in ipsilateral breast, 4.3% of patients received neoadjuvant hormone therapy (including aromatase inhibitor or tamoxifen), 11.4% received neoadjuvant chemotherapy, and 15.1% received adjuvant chemotherapy. There were 32.5% who were treated in prone position, 82.9% of patients had surgical clips placed at time of lumpectomy, mean interval from surgery to CT simulation was 54.1 ± 45.9 days, and mean lumpectomy cavity volume was 42.2 ± 52.0 cm^3^.

**Figure 1:**
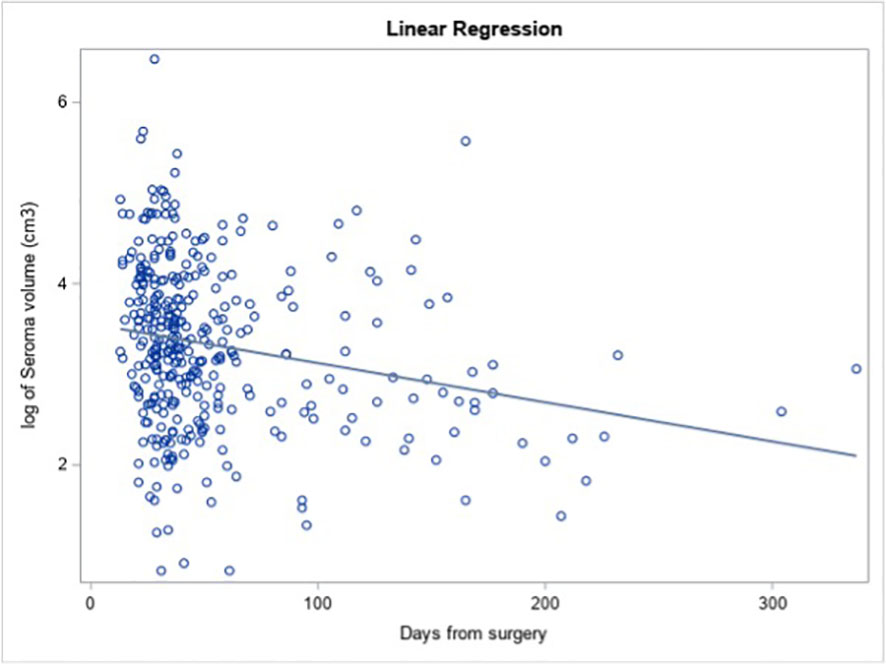
Mean lumpectomy cavity volume relative to interval after surgery.

Longer interval from surgery (as a continuous variable) was significantly associated with smaller lumpectomy cavity volume on univariate analysis (**Figure 1**, estimate = −0.006, p = 0.048). For each additional day after surgery, the log of the expected mean of lumpectomy cavity volume decreased by 0.006 cm^3^, meaning that a 0.6% decrease in lumpectomy cavity volume is expected per day.

On univariate analysis (**Table 2**), the factors that were significantly associated with lumpectomy cavity volume were interval after surgery, age, BMI, the receipt of neoadjuvant chemotherapy, race, hypertension, coronary artery disease, and position. Surgical re-excision, oncoplastic reduction, prior breast surgery, the receipt of chemotherapy, coronary artery disease, diabetes, the volume of excised breast tissue, smoking status, and the presence of clips were not significantly associated. Of note, 19 patients had missing BMI data and thus were not included in the multivariate model. On multivariate analysis (**Table 3**), the factors confirmed to have a significant effect on lumpectomy cavity volume (p < 0.05 for all) were the BMI, the receipt of neoadjuvant chemotherapy, prone position, hypertension, and race (Black people vs. White people). Higher BMI, the receipt of neoadjuvant chemotherapy vs. no neoadjuvant chemotherapy, prone position vs. supine, the presence of hypertension, and race (Black people vs. White people) were associated with larger mean lumpectomy cavity volume. For example, with the absence of hypertension, the log of lumpectomy cavity volume decreased by 0.305; the log of lumpectomy cavity volume increases with the presence of hypertension. With Black people compared to White people, the log of the lumpectomy cavity volume increased by 0.292.

**Table 2:** Univariate analysis results of lumpectomy cavity volume.

Univariate analysis	Estimate	P-value
Interval after surgery	-0.006	0.048
Maximal tumor diameter	0.066	0.1633
Volume of excised breast tissue	0.000	0.8847
Age	0.014	0.0209
BMI	0.030	<0.0001
Surgical re-excision (No vs. Yes)	0.048	0.8325
Oncoplastic reduction (No vs. Yes)	0.185	0.4934
Prior breast surgery (No vs. Yes)	0.085	0.7925
Neoadjuvant AI or tamoxifen (No vs. Yes)	-0.262	0.3078
Neoadjuvant chemotherapy (No vs. Yes)	-0.401	0.0096
Adjuvant chemotherapy (No vs. Yes)	0.391	0.1115
Race		0.0178
Race (Black people vs. White people)	0.337	0.0103
Race (others vs. White people)	-0.515	0.338
Diabetes mellitus (No vs. Yes)	-0.196	0.2038
Smoking status (current, former, or never)		0.4354
Smoking (current vs. never)	0.232	0.1989
Smoking (former vs. never)	0.041	0.7837
Hypertension (No vs. Yes)	-0.417	0.003
Coronary artery disease (No vs. Yes)	-0.441	0.0206
Position (prone vs. supine)	0.296	0.0223
Clips (No vs. Yes)	-0.353	0.1155

**Table 3:** Multivariate analysis results of lumpectomy cavity volume.

Multivariate analysis (N = 332)	Estimate	P-value
Interval after surgery	-0.006	0.0795
Age	0.012	0.0627
BMI	0.020	0.004
Race		0.3215
Race (Black people vs. White people)	0.292	0.0168
Race (others vs. White people)	-0.426	0.4383
Hypertension (No vs. Yes)	-0.305	0.0316
Position (prone vs. supine)	0.256	0.0347
Coronary artery disease (No vs. Yes)	-0.209	0.2893
Neoadjuvant chemotherapy (No vs. Yes)	-0.348	0.0172

Sensitivity analyses were done to assess the impact of prone vs. supine position (characteristics are separated by position in **Supplementary Table 1**). When separated into supine (n = 237) and prone (n = 114), longer interval from surgery (as a continuous variable) was not significantly associated with decreased lumpectomy cavity volume (**Supplementary Table 2**). On multivariate analysis of factors with lumpectomy cavity volume for patients in the supine position (**Supplementary Table 3**), race (Black people vs. White people), BMI, the receipt of neoadjuvant chemotherapy, and hypertension (presence vs. absence) had a significant effect on lumpectomy cavity volume as in the main analysis that did not separate the patients by position. For prone position, the presence of hypertension was significantly associated with larger mean lumpectomy cavity volume on multivariate analysis as in the main analysis, but race, BMI, and neoadjuvant chemotherapy were not. A regression analysis was also done to determine if hypertension was associated with other factors in this population, and this revealed that hypertension was associated with increased age, increased BMI, and having diabetes (**Supplementary Table 4**).

An additional analysis was done to assess the association between lumpectomy volume (cm^3^) and ipsilateral whole breast volume (cm^3^) and the BMI (**Supplementary Table 5A**). Following log transformations on both the predictor and outcome variables, a univariate regression model examined the relationship between the log of ipsilateral whole breast volume (cm^3^) and the log of lumpectomy volume (cm^3^) with repeated measures to account for patients with multiple lumpectomies. This demonstrated a positive relationship between the variables that was significant (p < 0.001) (**Supplementary Table 5B**). A univariate regression analysis examined the relationship between BMI and the log of ipsilateral whole breast volume (cm^3^) and demonstrated a positive relationship between the variables that was significant (p < 0.001) (**Supplementary Table 5C**).

## 4 Discussion

The growing literature discussed above on partial breast irradiation has shown overall that it is an effective and safe alternative to whole breast radiation in select patients. The ability to deliver this is predicated on reliable delineation of the lumpectomy cavity. In patients with larger lumpectomy cavities, the breast-to-target ratio is sometimes unfavorable for constraints used in major PBI trials. In those that still qualify for PBI, a geometrically larger treatment area is required for larger lumpectomy cavities. This study sought to describe factors that predict for larger lumpectomy cavity to aid in patient selection and identify modifiable changes that can optimize the target size prior to treatment planning.

Race, the presence of hypertension, higher BMI, the receipt of neoadjuvant chemotherapy, and prone position vs. supine remained significantly associated with larger mean lumpectomy cavity volume on multivariate analysis and thus were independently associated with lumpectomy cavity volume. Longer interval from surgery to simulation was associated with smaller lumpectomy cavity volume on univariate analysis and trended toward significance on multivariate analysis. The trends in lumpectomy cavity volume over time demonstrated in the present study and prior studies support the recommendation to perform the planning CT scan for PBI ideally within 8 weeks after surgery (14, 15). A study by Kader et al. showed that during weeks 3–8 after BCS, the mean lumpectomy cavity volume decreased from 47 to 30 cm^3^, stabilized during weeks 9–14 (mean 21 weeks), and was involuted after 14 weeks (14). Prone positioning is thought to elongate the cavity due to the effect of gravity, congruent with the result from our study that this position was associated with larger mean lumpectomy cavity volume. Sensitivity analyses demonstrated that the presence of hypertension remained significantly associated with larger mean lumpectomy cavity volume when separate analyses were done for prone and supine patients and that hypertension is also associated with increased age, BMI, and diabetes. An additional univariate analysis also found that lumpectomy cavity volume was associated with ipsilateral whole breast volume.

In order for a surgical cavity or wound to heal properly, adequate blood supply is necessary; therefore, conditions that impair circulation and oxygenation can delay the healing process (16). High blood pressure, diabetes, advanced age, and tobacco use may thus contribute to delayed healing. Data are lacking on the healing of the postoperative cavity or lumpectomy cavity after BCS. A study by Prendergast et al. examined the association of clinical factors for the association of volumetric change of the tumor bed before and during radiation and did not find any association with clinical factors including patient age, weight, tobacco use, re-excision, the volume of tissue removed, initial breast volume, or the use of chemotherapy (17).

Studies of breast reconstruction patients have shown postoperative complications at higher rates in patients with specific comorbidities, but the specific complications are variable between studies (18). Hypertension was found to be an independent risk factor for perioperative complications in a review of 1,170 consecutive expander/implant reconstructions, with hypertension defined as elevated blood pressure requiring medical therapy and associated with twice the risk of complications compared to patients without hypertension (19). Hypertension has also been associated with delayed surgical complications in breast reconstruction patients (5).

The significance of race predicting larger lumpectomy cavity volume is not clear. Multivariable analysis attempts to correct for imbalances in confounding variables examined here, and yet race remains a significant independent factor. Unmeasured social determinants of health may contribute to this difference, which bears further investigation. Race/ethnicity has been associated with differences in time to breast cancer diagnosis after suspicious breast abnormality first identified by a physical exam, mammogram, or ultrasound. Non-Hispanic Black people and Hispanic people were found to have a longer time to diagnosis than non-Hispanic White people, even with private health insurance (20). In this study, however, the patients all had early-stage breast cancer suitable for lumpectomy; thus, this is less likely to have been a factor. Potentially, the differences in lumpectomy cavity size could be related to differences in unexplored factors related to social determinants of health such as follow-up after surgery or differences in postoperative instructions.

Limitations of this study are related to its retrospective design. Additionally, the delineation of lumpectomy cavity can be subjective, especially in patients with dense breasts, and the analyzed dataset represented a variety of practitioners from the same institution. The delineation of lumpectomy cavity can especially be more difficult in the setting of oncoplastic reduction, which made up a small proportion of the patients in this dataset. Different surgeons were also involved in the cases that could be associated with variation in technique in addition to variation in the placement of surgical clips. Postoperative care patterns may have varied. The interpretation of results of continuous clinical variables may be harder to translate to clinical settings.

Especially relevant for patients planned to receive PBI, these data may be used to select patients for which longer time to simulation may result in smaller lumpectomy cavity volumes and therefore smaller PBI target volumes. Larger datasets and prospective evaluation would be ideal to confirm these hypotheses.

## Data availability statement

The raw data supporting the conclusions of this article will be made available by the authors, without undue reservation.

## Ethics statement

Consent was not required for this study. This study involved the secondary use of private information from the electronic medical record and was approved as exempt by the Indiana University institutional review board.

## Author contributions

Study design: AL and RR. Data collection: AL and FA. Data analysis: AL, LB, MS, RZ and RR. Manuscript draft writing: AL and RR. Manuscript review and approval: AL, FA, LB, MS, RZ and RR. All authors contributed to the article and approved the submitted version.
