# Supplementary material for: Patient characteristics and clinical factors affecting lumpectomy cavity volume: implications for partial breast irradiation
Source: Front Oncol. 2023 May 23;13:1118713. doi: 10.3389/fonc.2023.1118713 (PMC10242063; doi:10.3389/fonc.2023.1118713)
Supplement: Supplementary file 1 [file Table_1.docx]

**Supplementary Data**

**Supplementary Table 1a. Patient, tumor, and treatment characteristics separated by position**

|  | **Supine Position** | | | **Prone Position** | | |
| --- | --- | --- | --- | --- | --- | --- |
| **Characteristic** | **N** | **Mean ± SD** | **Median**  **(Q1 - Q3)** | **N** | **Mean ± SD** | **Median**  **(Q1 - Q3)** |
| Age (y)  **Mean ± Standard** | 237 | 60.0 ± 10.5 | 61.0  (53.0 - 67.0) | 114 | 61.6 ± 10.9 | 62.5  (55.0 - 70.0) |
| BMI (kg/m^2^)  **Mean ± Standard** | 227 | 31.0 ± 7.5 | 30.0  (25.1 - 35.7) | 105 | 31.4 ± 6.8 | 30.7  (26.8 - 34.3) |
| Days from surgery  **Median (Q1 - Q3)** | 237 | 55.7 ± 47.9 | 37.0  (28.0 - 57.0) | 114 | 50.9 ± 41.3 | 37.0  (29.0 - 57.0) |
| Lumpectomy volume (cm3)  **Median (Q1 - Q3)** | 237 | 38.0 ± 53.2 | 24.7  (14.2 - 45.0) | 114 | 51.0 ± 48.4 | 35.6  (18.1 - 63.5) |
| Total volume excised (cm3)  **Median (Q1 - Q3)** | 234 | 193.1 ± 366.8 | 94.5  (53.5 - 170.5) | 111 | 139.0 ± 193.0 | 88.8  (63.3 - 140.2) |
| Tumor size (max dimension  of largest tumor, cm)  **Median (Q1 - Q3)** | 237 | 1.3 ± 1.2 | 1.1  (0.6 - 1.8) | 114 | 1.3 ± 1.0 | 1.2  (0.7 - 1.7) |

**Supplementary Table 1b. Patient, tumor, and treatment characteristics separated by position**

|  |  | **Supine Position** | **Prone Position** |
| --- | --- | --- | --- |
| Site | IU | 44 (18.6%) | 31 (27.2%) |
|  | IU+ESK | 34 (14.3%) | 10 (8.8%) |
|  | MH | 22 (9.3%) | 12 (10.5%) |
|  | MH+ESK | 4 (1.7%) | 7 (6.1%) |
|  | NORTH | 28 (11.8%) | 27 (23.7%) |
|  | NORTH+ESK | 1 (0.4%) | 0 (0.0%) |
|  | WEST | 103 (43.5%) | 25 (21.9%) |
|  | WEST+ESK | 1 (0.4%) | 2 (1.8%) |
| Surgical re-excision | No | 215 (90.7%) | 101 (88.6%) |
|  | Yes | 22 (9.3%) | 13 (11.4%) |
| Oncoplastic reduction | No | 211 (89.0%) | 109 (95.6%) |
|  | Yes | 26 (11.0%) | 5 (4.4%) |
| Prior breast surgery | No | 225 (94.9%) | 108 (94.7%) |
|  | Yes | 12 (5.1%) | 6 (5.3%) |
| Neoadjuvant AI or tamoxifen | No | 226 (95.4%) | 110 (96.5%) |
|  | Yes | 11 (4.6%) | 4 (3.5%) |
| Neoadjuvant chemotherapy | No | 207 (87.3%) | 104 (91.2%) |
|  | Yes | 30 (12.7%) | 10 (8.8%) |
| Adjuvant chemotherapy | No | 199 (84.0%) | 99 (86.8%) |
|  | Yes | 38 (16.0%) | 15 (13.2%) |
| Race | Black | 55 (23.2%) | 29 (25.4%) |
|  | Other | 14 (5.9%) | 3 (2.6%) |
|  | White | 168 (70.9%) | 82 (71.9%) |
| Diabetes Mellitus | No | 189 (79.7%) | 102 (89.5%) |
|  | Yes | 48 (20.3%) | 12 (10.5%) |
| Smoking status | Current | 26 (11.0%) | 16 (14.0%) |
|  | Former | 72 (30.4%) | 33 (28.9%) |
|  | Never | 139 (58.6%) | 65 (57.0%) |
| Hypertension | No | 110 (46.4%) | 58 (50.9%) |
|  | Yes | 127 (53.6%) | 56 (49.1%) |
| Coronary artery disease | No | 221 (93.2%) | 109 (95.6%) |
|  | Yes | 16 (6.8%) | 5 (4.4%) |
| Clips | No | 44 (18.6%) | 16 (14.0%) |
|  | Yes | 193 (81.4%) | 98 (86.0%) |

**Supplementary Table 2. Variations in seroma characteristics relative to interval after surgery separated by position**

|  | ***Supine Position*** | | | ***Prone Position*** | | |
| --- | --- | --- | --- | --- | --- | --- |
| **Characteristic** | ***N*** | ***Estimate*** | ***p*** | ***N*** | ***Estimate*** | ***p*** |
| Seroma volume (cm^3^) | 237 | -0.005 | 0.158 | 114 | -0.007 | 0.139 |

**Supplementary Table 3a. Univariate analysis results of seroma volume separated by position**

|  | **Supine Position** | | | **Prone Position** | | |
| --- | --- | --- | --- | --- | --- | --- |
| **Univariate analysis** | **Estimate** | **Exp**  **(Estimate)** | **p-value** | **Estimate** | **Exp**  **(Estimate)** | **p-value** |
| Interval after surgery | -0.005 | 0.995 | 0.158 | -0.007 | 0.993 | 0.139 |
| Maximal tumor diameter | 0.049 | 1.050 | 0.457 | 0.158 | 1.172 | 0.063 |
| Volume of excised breast tissue | 0.000 | 1.000 | 0.893 | 0.000 | 1.000 | 0.343 |
| Age | 0.009 | 1.009 | 0.279 | 0.018 | 1.018 | 0.019 |
| BMI | 0.035 | 1.035 | <0.001 | 0.023 | 1.023 | 0.019 |
| Surgical re-excision  (No vs Yes) | -0.024 | 0.976 | 0.937 | 0.188 | 1.207 | 0.560 |
| Oncoplastic reduction  (No vs Yes) | 0.005 | 1.005 | 0.987 | 0.771 | 2.163 | 0.386 |
| Prior breast surgery  (No vs Yes) | -0.025 | 0.975 | 0.950 | 0.280 | 1.323 | 0.584 |
| Neoadjuvant AI or tamoxifen  (No vs Yes) | 0.293 | 1.341 | 0.604 | -0.943 | 0.389 | <0.001 |
| Neoadjuvant chemotherapy  (No vs Yes) | -0.570 | 0.565 | 0.002 | -0.085 | 0.919 | 0.771 |
| Adjuvant chemotherapy  (No vs Yes) | 0.232 | 1.261 | 0.427 | 0.699 | 2.013 | 0.127 |
| Race |  |  |  |  |  |  |
| Race (black vs white) | 0.443 | 1.557 | 0.013 | 0.160 | 1.174 | 0.389 |
| Race (other vs white) | -0.384 | 0.681 | 0.531 | -0.720 | 0.487 | 0.532 |
| Diabetes Mellitus (No vs Yes) | -0.244 | 0.784 | 0.218 | -0.258 | 0.773 | 0.276 |
| Smoking current/former/never (c/f/n) |  |  |  |  |  |  |
| Smoking c/f/n (Current vs Never) | 0.446 | 1.562 | 0.043 | -0.165 | 0.848 | 0.594 |
| Smoking c/f/n (Former vs Never) | -0.047 | 0.954 | 0.829 | 0.171 | 1.187 | 0.358 |
| Hypertension (No vs Yes) | -0.401 | 0.669 | 0.041 | -0.473 | 0.623 | 0.010 |
| Coronary artery disease (No vs Yes) | -0.562 | 0.570 | 0.014 | -0.239 | 0.788 | 0.493 |
| Clips (No vs Yes) | -0.270 | 0.763 | 0.336 | -0.465 | 0.628 | 0.201 |

**Supplementary Table 3b. Multivariate analysis results of seroma volume – supine**

|  | **Supine (N=227)** | | |
| --- | --- | --- | --- |
| **Multivariate analysis** | **Estimate** | **Exp(estimate)** | **P-value** |
| BMI | 0.111 | 1.117 | <0.001 |
| Race |  |  | <0.001 |
| Race (black vs white) | 0.892 | 2.440 | <0.001 |
| Race (other vs white) | -0.479 | 0.619 | 0.591 |
| Hypertension (No vs Yes) | -0.738 | 0.478 | 0.002 |
| Coronary artery disease (No vs Yes) | -0.134 | 0.875 | 0.455 |
| Neoadjuvant chemotherapy (No vs Yes) | -1.633 | 0.195 | <0.001 |
| Smoking c/f/n |  |  | <0.001 |
| Smoking c/f/n (Current vs. Never) | 1.039 | 2.826 | <0.001 |
| Smoking c/f/n (Former vs. Never) | -1.974 | 0.139 | <0.001 |

**Supplementary Table 3c. Multivariate analysis results of seroma volume – prone**

|  | **Prone (N=105)** | | |
| --- | --- | --- | --- |
| **Multivariate analysis** | **Estimate** | **Exp(estimate)** | **P-value** |
| Maximal tumor diameter | 0.127 | 1.135 | 0.075 |
| Age | 0.008 | 1.008 | 0.306 |
| BMI | 0.012 | 1.012 | 0.255 |
| Hypertension (No vs Yes) | -0.511 | 0.600 | 0.009 |
| Neoadjuvant AI or tamoxifen  (No vs Yes) | -1.233 | 0.291 | <0.001 |

**Supplementary Table 4a. Univariate logistic analysis of hypertension status**

|  | **Hypertension** | | |  |
| --- | --- | --- | --- | --- |
| **Variable** | **Overall N=351** | **Yes N=183** | **No N=168** | **p-value** |
|  |  |  |  |  |
| Days from surgery | 37.0 (28.0, 57.0) | 38.0 (29.0, 56.0) | 37.0 (28.0, 57.5) | 0.61 |
| Lumpectomy volume (cm3) | 26.7 (14.8, 53.9) | 30.2 (17.6, 59.8) | 25.0 (12.1, 43.0) | 0.003 |
| Tumor size (max dimension of  largest tumor, cm) | 26.7 (14.8, 53.9) | 30.2 (17.6, 59.8) | 25.0 (12.1, 43.0) | 0.38 |
| Total volume excised (cm3) | 89.6 (58.5, 161.3) | 107.0 (63.3, 180.1) | 80.5 (52.9, 122.3) | 0.004 |
| Age | 60.54 ± 10.63 | 64.34 ± 9.16 | 56.39 ± 10.60 | <.0001 |
| BMI | 31.10 ± 7.26 | 32.87 ± 7.09 | 29.11 ± 6.95 | <.0001 |
| Surgical Re-excision |  |  |  | 0.66 |
| - No | 316 (90.0%) | 166 (90.7%) | 150 (89.3%) |  |
| - Yes | 35 (10.0%) | 17 (9.3%) | 18 (10.7%) |  |
| Oncoplastic reduction |  |  |  | 0.49 |
| - No | 320 (91.2%) | 165 (90.2%) | 155 (92.3%) |  |
| - Yes | 31 (8.8%) | 18 (9.8%) | 13 (7.7%) |  |
| Prior breast surgery |  |  |  | 0.10 |
| - No | 333 (94.9%) | 177 (96.7%) | 156 (92.9%) |  |
| - Yes | 18 (5.1%) | 6 (3.3%) | 12 (7.1%) |  |
| Neoadjuvant AI |  |  |  | 0.53 |
| - No | 336 (95.7%) | 174 (95.1%) | 162 (96.4%) |  |
| - Yes | 15 (4.3%) | 9 (4.9%) | 6 (3.6%) |  |
| Neoadjuvant chemotherapy |  |  |  | 0.96 |
| - No | 311 (88.6%) | 162 (88.5%) | 149 (88.7%) |  |
| - Yes | 40 (11.4%) | 21 (11.5%) | 19 (11.3%) |  |
| Adjuvant chemotherapy |  |  |  | 0.68 |
| - No | 298 (84.9%) | 154 (84.2%) | 144 (85.7%) |  |
| - Yes | 53 (15.1%) | 29 (15.8%) | 24 (14.3%) |  |
| Race |  |  |  | 0.09 |
| - Black | 84 (23.9%) | 51 (27.9%) | 33 (19.6%) |  |
| - Other | 17 (4.8%) | 6 (3.3%) | 11 (6.5%) |  |
| - White | 250 (71.2%) | 126 (68.9%) | 124 (73.8%) |  |
| DM |  |  |  | <.0001 |
| - No | 291 (82.9%) | 130 (71.0%) | 161 (95.8%) |  |
| - Yes | 60 (17.1%) | 53 (29.0%) | 7 (4.2%) |  |
| Smoking c/f/n |  |  |  | 0.11 |
| - Current | 42 (12.0%) | 18 (9.8%) | 24 (14.3%) |  |
| - Former | 105 (29.9%) | 63 (34.4%) | 42 (25.0%) |  |
| - Never | 204 (58.1%) | 102 (55.7%) | 102 (60.7%) |  |
| Coronary artery disease |  |  |  | 0.002 |
| - No | 330 (94.0%) | 165 (90.2%) | 165 (98.2%) |  |
| - Yes | 21 (6.0%) | 18 (9.8%) | 3 (1.8%) |  |
| Clips |  |  |  | 0.84 |
| - No | 60 (17.1%) | 32 (17.5%) | 28 (16.7%) |  |
| - Yes | 291 (82.9%) | 151 (82.5%) | 140 (83.3%) |  |
| Position |  |  |  | 0.43 |
| - Prone | 114 (32.5%) | 56 (30.6%) | 58 (34.5%) |  |
| - Supine | 237 (67.5%) | 127 (69.4%) | 110 (65.5%) |  |

**Supplementary Table 4b. Multivariate logistic analysis of hypertension status**

| **Multivariate analysis** | **Estimate** | **Odds Ratio** | **p-value** |
| --- | --- | --- | --- |
| Lumpectomy volume (cm3) | 0.005 | 1.005 | 0.17 |
| Total volume excised (cm3) | 0.000 | 1.000 | 0.28 |
| Age | 0.079 | 1.082 | <.0001 |
| BMI | 0.067 | 1.069 | 0.001 |
| Race |  |  | 0.68 |
| Race (Black vs. White) | 0.279 | 1.322 | 0.38 |
| Race (Other Vs. White) | 0.083 | 1.087 | 0.90 |
| Diabetes Mellitus (No vs. Yes) | -1.792 | 0.167 | <.0001 |
| Coronary artery disease (No vs. Yes) | -0.904 | 0.405 | 0.19 |

**Supplementary Table 5a. Patient and tumor characteristics for breast volume measures**

| **Characteristic** | **N** | **Mean ± SD** | **Median (Q1 - Q3)** |
| --- | --- | --- | --- |
| BMI (kg/m^2^) **mean ± standard** | 332 | 31.1 ± 7.3 | 30.4 (25.5 - 35.2) |
| Ipsilateral whole breast volume (cm3) **Median (Q1 - Q3)** | 357 | 1,169.2 ± 630.9 | 1,070.5 (697.9 – 1,534.6) |
| Lumpectomy volume (cm3) **Median (Q1 - Q3)** | 357 | 42.2 ± 51.7 | 26.7 (14.8 - 53.9) |

**Supplementary Table 5b. Analysis of ipsilateral whole breast volume and lumpectomy volume with repeated measures (n=357)**

| **Characteristic** | **Estimate** | **95% CI** | **Exp(Estimate)** | **p-value** |
| --- | --- | --- | --- | --- |
| Log of Ipsilateral whole breast volume, cm3 | 0.579 | (0.433, 0.724) | 1.784 | **< 0.001** |

**Supplementary Table 5c. Analysis of body mass index (BMI) and ipsilateral whole breast volume (n=332)**

| **Characteristic** | **Estimate** | **95% CI** | **Exp (Estimate)** | **p-value** |
| --- | --- | --- | --- | --- |
| BMI | 0.055 | (0.048, 0.061) | 1.057 | **< 0.001** |
